# Vitreomacular Traction and Outer Retinal Structural Changes

**DOI:** 10.4274/tjo.galenos.2018.79577

**Published:** 2019-04-30

**Authors:** Şeyda Yıldırım, Jale Menteş, Mine Barış

**Affiliations:** 1 Adıyaman University Training and Research Hospital, Ophthalmology Clinic, Adıyaman, Turkey; 2 Ege University Faculty of Medicine, Department of Ophthalmology, İzmir, Turkey; 3 Buca Seyfi Demirsoy State Hospital, Ophthalmology Clinic, İzmir, Turkey

**Keywords:** Vitreomacular traction, outer retinal microhole, optical coherence tomography

## Abstract

In this case report we aimed to present the outer retinal structural changes secondary to vitreomacular traction (VMT). Outer retinal structural changes occurring secondary to VMT due to incomplete posterior vitreous detachment were described retrospectively with spectral-domain optical coherence tomography in 3 eyes of 3 patients. The patients ranged in age from 58 to 65 years and best corrected visual acuity in the 3 eyes was 4/10, 8/10, and 9/10. All of the patients were symptomatic and exhibited outer retinal microholes at the fovea extending from the retinal pigment epithelium to outer limiting membrane, with an overlying operculum on the detached posterior hyaloid membrane over the macula following spontaneous resolution of VMT. In the mean follow-up period of 32 months, the outer retinal microholes decreased in size but did not completely resolve. As demonstrated in these cases, VMT can cause small outer retinal layer defects without signs of full-thickness macular hole. These lesions can cause symptoms and affect visual function, and may be permanent structural changes.

## Introduction

Posterior vitreous detachment (PVD) is the separation of the posterior vitreous cortex from the internal limiting membrane due to liquefaction of the vitreous gel and weakened vitreoretinal adhesion. This detachment process occurs in four stages. Stage 1 involves perifoveal separation of the vitreous with adhesion to the fovea, midperipheral retina, and optic disc. Stage 2 is characterized by complete vitreofoveal separation with persistent adhesion to the midperipheral retina and optic disc. In Stage 3, there is vitreous detachment from the midperipheral retina with persistent adhesion to the optic disc. In Stage 4, also known as complete PVD, the vitreous is completely detached from the optic disc as well.

Various clinical manifestations may be encountered as a result of tractional interactions between the vitreous and retina during progression of PVD. One of these is vitreomacular traction (VMT) syndrome. Vitreomacular traction during the PVD process can cause structural defects such as cysts in the inner and/or outer retinal layers, full-thickness macular hole, and schisis, or can regress spontaneously without causing any structural changes in the retinal layers.^1^ In VMT syndrome, visual symptoms such as metamorphopsia, scotoma, and decreased visual acuity may arise due to anteroposterior traction exerted on the macula. Although the pathogenesis of the disease has not been fully elucidated, the ability to visualize the retina and vitreoretinal interface in high resolution with spectral domain optic coherence tomography (SD-OCT) has provided a better understanding of the effects of tractional forces on the retina and macular surface.^1^

In this article, we present three eyes of three patients with microdefects that developed secondary to VMT in the outer retinal layers only, without causing any anatomic abnormality in the inner retinal layers, together with their follow-up results.

## Case Reports

### Case 1

**Figure 1:**
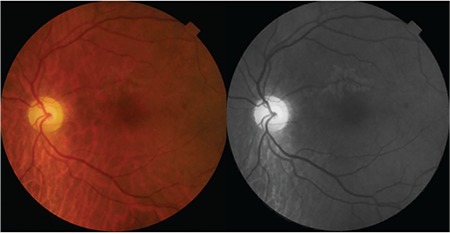
Case 1: Color fundus and red-free fundus images

**Figure 2:**
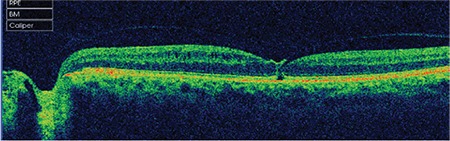
Case 1: Spectral domain optical coherence tomography shows a microdefect in the outer retinal layers at the fovea due to vitreomacular traction

A 58-year-old woman presented with complaints of decreased visual acuity and metamorphopsia in her left eye. Her history was unremarkable and full ophthalmological examination was normal. Color fundus and red-free fundus images (Topcon SD-OCT; Topcon Medical Systems, Paramus, New Jersey, USA) were normal (Figure 1). Best corrected visual acuity (BCVA) of her left eye was 8/10 and SD-OCT (Topcon SD-OCT; Topcon Medical Systems, Paramus, NJ, USA) revealed incomplete PVD and VMT. There was puckering and disorganization of the inner retinal layers due to anteroposterior traction, irregular foveal contour, and a defect approximately 140 microns wide in the external limiting membrane (ELM) and photoreceptor inner segment–outer segment (IS/OS) layers (Figure 2).

**Figure 3:**
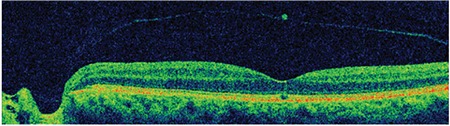
Patient 1: At 5-month follow-up, spectral domain optical coherence tomography shows a defect in the external limiting membrane and inner segment/ outer segment line and an operculum on the detached posterior hyaloid membrane

**Figure 4:**
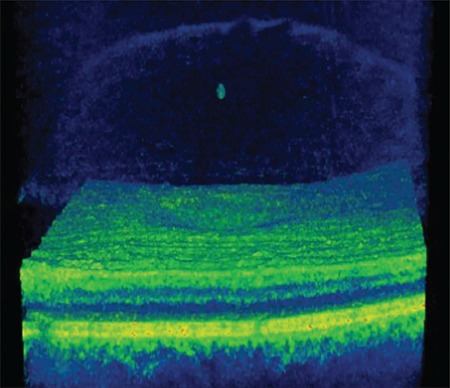
Patient 1: Three-dimensional spectral domain optical coherence tomography shows operculum over the macula on the detached posterior hyaloid membrane

After 5 months of follow-up, the VMT spontaneously regressed, after which the patient’s metamorphopsia resolved suddenly, BCVA in that eye increased to 9/10, and SD-OCT revealed complete normalization of the foveal contour as well as regression of the irregularities in the inner retinal folds. In addition, an operculum was observed over the macula attached to the residual posterior hyaloid membrane, and a defect 90 microns in diameter persisted in the ELM and IS/OS layers (Figure 3). At 46-month follow-up, the patient was asymptomatic and the defect in the outer retinal layers was found to persist at a size of 68 microns. The operculum on the detached posterior hyaloid membrane over the macula was also visualized using three-dimensional (3D) SD-OCT (Figure 4).

### Case 2

**Figure 5:**
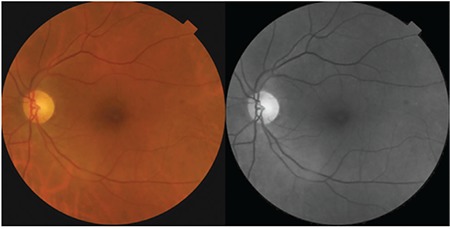
Patient 2: Color fundus and red-free fundus images

**Figure 6:**
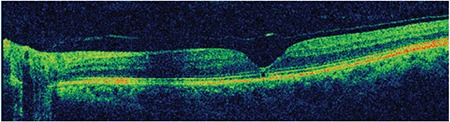
Patient 2: Spectral domain optical coherence tomography shows a microdefect in the external limiting membrane and inner segment/outer segment line and an operculum on the detached posterior hyaloid membrane

**Figure 7:**
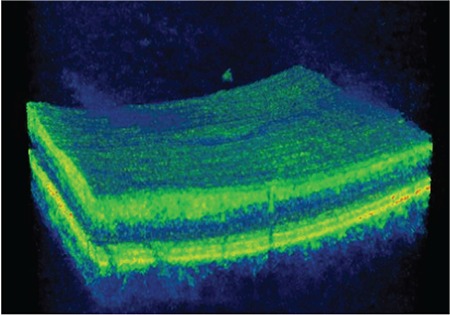
Patient 2: Three-dimensional spectral domain optical coherence tomography shows operculum over the macula on the detached posterior hyaloid membrane

A 65-year-old woman presented due to sudden-onset metamorphopsia in her left eye. BCVA was 4/10 in the affected eye and color fundus and red-free fundus images (Topcon SD-OCT; Topcon Medical Systems, Paramus, New Jersey, USA) were normal (Figure 5). Examination of the left eye with SD-OCT (Topcon SD-OCT; Topcon Medical Systems, Paramus, NJ, USA) revealed grade 3 PVD and an operculum over the macula, as well as a 156-micron outer retinal defect in the ELM and IS/OS (Figure 6). The operculum on the residual posterior hyaloid membrane over the macula was also observed in 3D SD-OCT (Figure 7).

**Figure 8:**
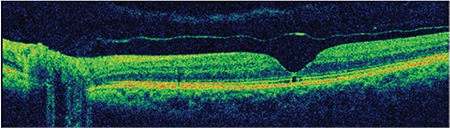
Patient 2: At 42-month follow-up, spectral domain optical coherence tomography showed a microdefect at the external limiting membrane and inner segment/outer segment line

At 42-month follow-up, BCVA in the affected eye was 6/10 and a 90-micron defect persisted in the ELM and IS/OS (Figure 8).

### Case 3

**Figure 9:**
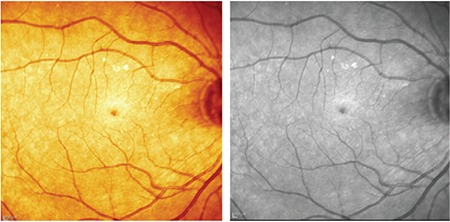
Patient 3: Infrared images

**Figure 10:**
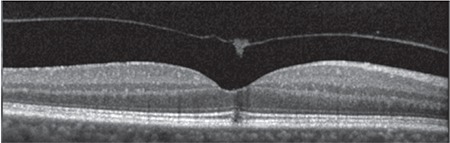
Patient 3: Spectral domain optical coherence tomography shows a microdefect in the external limiting membrane and inner segment/outer segment line and an operculum on the detached posterior hyaloid membrane

A 59-year-old man presented due to central scotoma that had recently developed in his right eye. BCVA was 8/10 and infrared fundus images (Heidelberg Spectralis HRA+OCT; Heidelberg Engineering, Heidelberg, Germany) showed a dark spot in the fovea (Figure 9). SD-OCT (Heidelberg Spectralis HRA+OCT; Heidelberg Engineering, Heidelberg, Germany) revealed grade 3 PVD with an operculum attached to the posterior hyaloid membrane remnants overlying the macula, and a 122-micron defect in the ELM and IS/OS layers (Figure 10).

**Figure 11:**
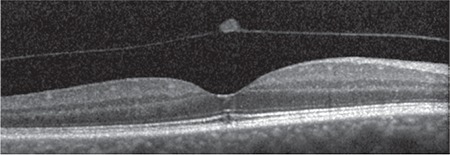
Patient 3: At 10-month follow-up, spectral domain optical coherence tomography shows the persistent microdefect in the external limiting membrane and inner segment/outer segment line

After a 10-month follow-up period, the patient had BCVA of 9/10, persistent central scotoma, and the outer retinal defect was unchanged (Figure 11).

## Discussion

In this paper, we present outer lamellar microdefects appearing as disruptions in the continuity of the ELM and IS/OS layers of the retina in OCT in three eyes, two of which were detected after the regression of VMT and the other that was observed both concurrently with and after regression of VMT, as well as their outcomes over a mean follow-up period of 32 months.

Although VMT associated with incomplete PVD is known to frequently cause changes in the inner retinal folds, it was also reported to lead to the development of a rectangular outer retinal defect in the outer macular layers, and these microdefects were referred to as microholes. These outer retinal microholes are defects extending from the inner boundary of the retinal pigment epithelium (RPE) to the outer limiting membrane, including the photoreceptor IS/OS junction.^2^ Emerson et al.^3^ claimed that the term “microhole”, which was coined before OCT, was not accurate to the actual anatomy of these lesions, and that “microcyst” would be a more appropriate term for these small defects of the outer retinal layer at the fovea. In the following years, there was an increase in the number of case reports and studies attempting to explain the etiology of the new clinical entity of outer lamellar microholes.

Although vitreofoveal traction and trauma are known to trigger the formation of microholes, we have yet to identify all of the etiologic factors.^4^ Various studies have reported that they can occur either with or without vitreomacular tractional interactions. In their study investigating 14 macular microholes, Ooto et al.^5^ reported that 64% of the eyes (9 eyes) exhibited complete or incomplete PVD, while 36% (5 eyes) showed no signs of PVD or vitreous traction. They suggested the possibility of a primary pathology in this group of microholes that may arise in the photoreceptor layer. Other authors have reported that outer retinal holes may occur as a result of vitreomacular traction as well as a wide range of other conditions such as trauma, solar maculopathy, welder’s and tamoxifen maculopathy, juxtafoveal macular telangiectasia, achromatopsia, alkyl nitrate abuse, acute retinal pigment epithelitis, and early Stargardt’s disease.^2^

In their report of a single case, Lai et al.^6^ detected a full-thickness macular microhole 137 microns in diameter in a patient who had PVD with an operculum on the detached posterior hyaloid membrane, and suggested that this microhole formed when a small piece detached from the fovea due to acute anteroposterior vitreous traction. At 3-week follow-up, they observed apposition of the full-thickness defect in the inner retinal layers, while a small defect persisted in the outer retina at the IS/OS border. Thus the authors reported that outer lamellar microdefects can be seen during the spontaneous resolution of full-thickness defects.

Yu et al.^7^ demonstrated oblique traction exerted on the fovea by incomplete PVD using swept-source OCT, and presented the multimodal imaging characteristics of a 38-micron defect in the continuity of the outer retinal layers in the photoreceptor region of the fovea. They used the term “foveal red spot syndrome” to describe this lesion.

Takahashi et al.^8^ reported that photoreceptor layer elevation associated with perifoveal PVD can cause the formation of outer retinal microholes.

In all three of our cases, VMT was determined to be the cause of the microdefects in the outer retinal layers. This conclusion was supported by our observations of puckering in all retinal layers and irregular foveal contour due to traction as well as the microdefect that appeared in the ELM and IS/OS layers during our 5-month follow-up of the VMT process in our first case. We believe that the opercula that appeared directly over the fovea on the floating posterior hyaloid membrane after VMT regression and persisted throughout follow-up were formed by detachment of a piece of the outer retinal layer.

Of the publications in the literature describing the characteristics of microdefects (microholes) in the outer retinal layers after VMT, only two have mentioned the presence of foveal operculum, in one case each.^9,10^ Similar opercula are known to form over the fovea in full-thickness macular holes caused by VMT, and histopathological examination of samples showed that these opercula include photoreceptor cells.^9,10^

In addition to OCT studies on microholes, there are also microperimetry studies in the literature. Gella et al.^11^ evaluated OCT and microperimetry in 12 eyes with defects in the photoreceptor layer at the IS/OS line. They reported that the mean diameter of microholes was 163±99 μm, retinal sensitivity was reduced in the area corresponding to the hole and had a mean of 13.79±4.6 dB, and that microhole diameter was negatively correlated with retinal sensitivity.

With advances in technology, more studies will provide increasingly detailed information. Especially with the widespread use of high-resolution OCT devices, it has been shown that microdefects involving only the outer retinal layers can develop in the fovea. These defects, which appear on OCT as a discontinuity in the ELM and IS/OS layers of the outer retina under the fovea (outer retinal microholes), can arise both during VMT and after resolution of VMT, as seen in our cases. We believe that future studies on these microholes will yield more information on the etiology and natural history of the disease.
